# Implications of fasting plasma glucose variability on the risk of incident peripheral artery disease in a population without diabetes: a nationwide population-based cohort study

**DOI:** 10.1186/s12933-022-01448-1

**Published:** 2022-01-31

**Authors:** Hye Soo Chung, Soon Young Hwang, Jung A. Kim, Eun Roh, Hye Jin Yoo, Sei Hyun Baik, Nan Hee Kim, Ji A. Seo, Sin Gon Kim, Nam Hoon Kim, Kyung Mook Choi

**Affiliations:** 1 Division of Endocrinology and Metabolism, Department of Internal Medicine, Kangnam Sacred Heart Hospital, College of Medicine, Hallym University, Seoul, South Korea; 2 Department of Biostatistics, College of Medicine, Korea University, South Seoul, South Korea; 3 Division of Endocrinology and Metabolism, Department of Internal Medicine, College of Medicine, Korea University, Seoul, South Korea; 4 Division of Endocrinology and Metabolism, Department of Internal Medicine, Korea University Guro Hospital, 80 Guro-Dong, Guro-Gu, Seoul, 08308 South Korea

**Keywords:** Glycemic variability, Fasting plasma glucose, Peripheral artery disease

## Abstract

**Background:**

Diabetes have been known as a traditional risk factor of developing peripheral artery disease (PAD). However, the study evaluating the impact of long-term glycemic variability on the risk of developing PAD is limited, especially in a general population without diabetes.

**Methods:**

We included 152,931 individuals without diabetes from the Korean National Health Insurance Service–Health Screening Cohort. Fasting plasma glucose (FPG) variability was measured using coefficient variance (FPG-CV), standard deviation (FPG-SD), and variability independent of the mean (FPG-VIM).

**Results:**

A total of 16,863 (11.0%) incident cases of PAD were identified during a median follow-up of 8.3 years. Kaplan–Meier curves showed a progressively increasing risk of PAD in the higher quartile group of FPG variability than in the lowest quartile group (log rank *P* < 0.001). Multivariable Cox proportional hazard analysis showed the hazard ratio for PAD prevalence as 1.11 (95% CI 1.07–1.16, *P* < 0.001) in the highest FPG-CV quartile than in the lowest FPG-CV quartile after adjusting for confounding variables, including mean FPG. Similar degree of association was shown in the FPG-SD and FPG-VIM. In sensitivity analysis, the association between FPG variability and the risk of developing PAD persisted even after the participants were excluded based on previously diagnosed diseases, including stroke, coronary artery disease, congestive heart failure, chronic kidney disease, or current smokers or drinkers. Subgroup analysis demonstrated that the effects of FPG variability on the risk of PAD were more powerful in subgroups of younger age, regular exercisers, and those with higher income.

**Conclusions:**

Increased long-term glycemic variability may have a significant prognostic effect for incident PAD in individuals without diabetes.

**Graphical Abstract:**

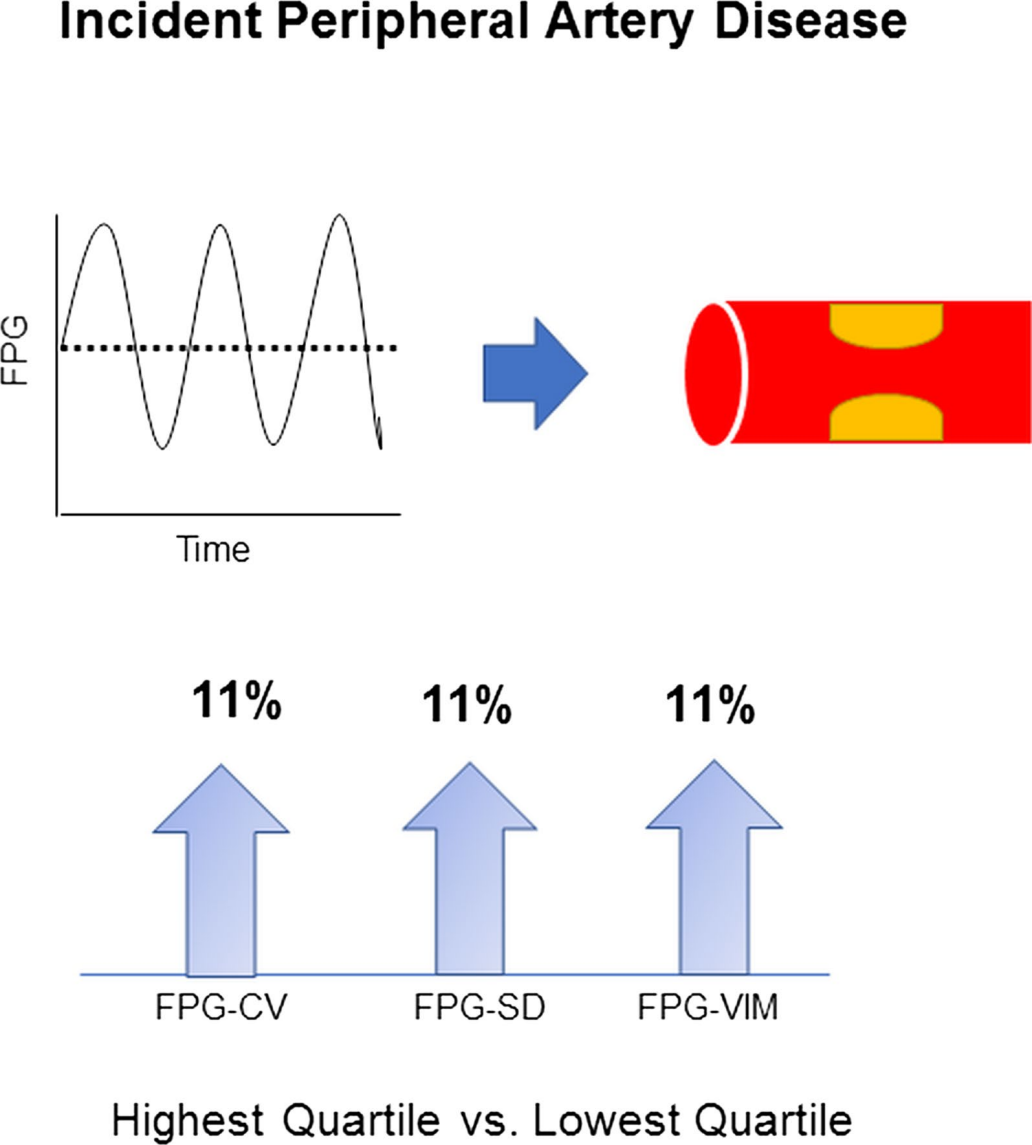

**Supplementary Information:**

The online version contains supplementary material available at 10.1186/s12933-022-01448-1.

## Background

Peripheral artery disease (PAD) is a chronic arterial occlusive disease caused by atherosclerosis, especially in the lower extremities [1]. Clinical expression is various including asymptomatic, intermittent claudication, resting pain, tissue damage, ulcers, and gangrene [2]. In the United States, among individuals aged > 40 years, the prevalence of PAD was reported at approximately 10.7% and severe PAD, such as severe limb ischemia, at 1.3% [3]. In addition, it has been reported that the prevalence of PAD increases with age and is accompanied by about 20% of the elderly population aged ≥ 80 years [4]. Despite its high prevalence, because people are still poorly informed regarding the potential risk factors, pathology, and clinical expression, PAD is generally underdiagnosed and undertreated. Moreover, patients with PAD have a higher incidence of cardiovascular disease (CVD) and CVD-related mortality [1, 5]; because both CVD and PAD are atherosclerosis-related ischemic artery diseases that share similar pathogenetic backgrounds. Given the cardiovascular burden, continuous investigation and efforts to reduce the risk factors are advisable.

Several traditional risk factors for atherosclerosis, such as smoking, diabetes, hypertension, and dyslipidemia, have been linked to an increased risk of developing PAD [2]. In particular, the population with diabetes had a 38–84% higher risk of PAD than the population without diabetes [6]. Furthermore, preceding observational studies have suggested that well-controlled glucose levels measured with fasting plasma glucose (FPG) or glycated hemoglobin (HbA1c) have a beneficial outcome on limb ischemia among patients with diabetes and PAD [7, 8]. Moreover, high levels of inflammation markers such as C-reactive protein (CRP) or Interleukin-6 are predictors for PAD development [2].

Recent evidence have proposed glycemic variability to be a novel target of glycemic control; thus, glycemic variability may be an important predictor of atherosclerosis independent of average glucose levels. It has been reported that long-term glycemic variability is positively associated with macrovascular/microvascular complications and mortality in individuals with diabetes [9–11]. In addition, several studies have suggested that increased glycemic fluctuations cause more atherosclerosis-related reactions by stimulating inflammatory cytokines, oxidative stress, endothelial dysfunction, and insulin resistance than sustained hyperglycemia [12, 13]. In vivo and in vitro studies have also revealed a relationship between glucose fluctuation and endothelial cell/endothelium damage [14–16]. Furthermore, we reported that visit-to-visit variability of FPG may be a significant predictor of chronic cardiometabolic and neurodegenerative diseases, such as type 2 diabetes [17], cardiovascular disease [18], non-alcoholic fatty liver disease [19], and Parkinson’s disease [20] in the Korean general population without diabetes.

In this study, we aimed to investigate the association between long-term glycemic variability and the risk of incident PAD in the general population without diabetes using a database from the longitudinal National Health Insurance Service-National Health Screening Cohort (NHIS-HEALS) of the Korean population.

## Methods

### Data source and study population

**Fig. 1:**
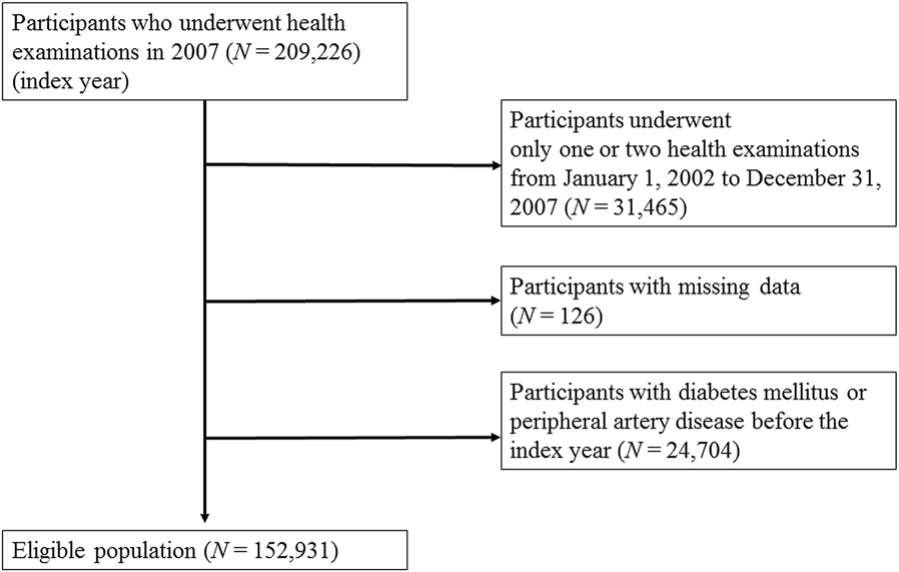
Flowchart for inclusion of the study population

The NHIS-HEALS is a randomly selected database that includes about 10% of the general population between 40 and 79 years old within the Korean NHIS database. The Korean NHIS is a government-managed mandatory public health insurance program that provides biennial national health examinations for the general population. The Korean NHIS comprises sociodemographic information (such as sex, age, and income), claim data (such as codes of diagnoses, pharmacy, and death), and a national health examination (such as anthropometric measurements, blood pressure, laboratory tests, and standardized self-reported questionnaires on smoking status, alcohol consumption, and exercise). Height, weight, and laboratory tests were done after an overnight fast, and the quality assurance process was supervised by the Korean Association of Laboratory Quality Control. The samples were initially collected from 209,226 participants who underwent a health examination in 2007 (index year). Participants were excluded based on the following criteria: those who underwent one or two health examinations from January 1, 2002, to December 31, 2007 (n = 31,465), those who had missing laboratory data for at least one variable (n = 126), and individuals who had previously been diagnosed with diabetes and PAD prior to 2007 (n = 24,704). Finally, a total of 152,931 participants were included in the analysis (Fig. 1).

These protocols were approved by the NHIS review committee. The Hallym University Institutional Review Board (IRB) approved the study protocol in accordance with the Declaration of Helsinki of the World Medical Association. Informed consent was waived because anonymous and unidentified information was used for the analysis.

### Definition of FPG variability

The visit-to-visit variability in FPG was defined using three or more FPG levels measured during serial health examinations conducted from January 1, 2002, to December 31, 2007. The number of FPG level measurements per participants were as follows: three (n = 75,688; 49.5%), four (n = 16,501; 10.8%), five (n = 22,521; 14.7%), and six (n = 38,221; 25.0%) measurements. Three methods of measurement were used for the calculation of FPG variability that included the coefficient variance (FPG-CV), standard deviation (FPG-SD), and variability independent of the mean (FPG-VIM). The CV was defined as SD/mean × 100, whereas VIM was defined as 100 × SD/Mean^β^, where β is the regression coefficient based on the natural logarithm of the SD divided by the natural logarithm of the mean.

### Measurements and definitions

The primary outcomes of this study were newly incident PAD that were identified after health examinations in 2007 (index year) until December 2015 or their date of death. PAD was diagnosed using the International Classification of Diseases, Tenth Revision (ICD-10) codes (I70.2, I70.9, I73.9, I74.3, and I74.4) [21] in both inpatient and outpatient settings. Diabetes was defined based on the criteria of fasting glucose level ≥ 7.0 mmol/L or by having at least one prescription claim per year for medication for diabetes under the ICD-10 codes E10–E14. Impaired fasting glucose (IFG) was defined as FPG levels between 5.6 mmol/L and 6.9 mmol/L among participants without diabetes. Hypertension was defined based on the criteria of systolic/diastolic blood pressure ≥ 140/90 mmHg or by having at least one prescription claim per year for hypertension medication under ICD-10 codes I10–I15. Dyslipidemia was defined based on the criteria of total cholesterol ≥6.2 mmol/L or by having at least one prescription claim per year for a dyslipidemia medication under ICD-10 code E78. Stroke diagnosis was defined as ICD-10 codes I60-I64 on the admission record with computerized tomography or magnetic resonance imaging claim data. The diagnosis of coronary artery disease (CAD) was defined as ICD-10 codes I50-I25 with coronary artery angiography (HA670). The diagnosis of chronic kidney disease (CKD) was defined as ICD-10 codes N18 or N19. The diagnosis of congestive heart failure (CHF) was defined as ICD-10 code I50 during hospitalization. Body mass index (BMI) was calculated as weight in kilograms divided by the square of height in meters (kg/m^2^). Smoking status and information concerning alcohol consumption were obtained from a questionnaire administered during the health examination. Regular exercise was defined as strenuous physical activity for at least 20 min or more than five times per week. Income levels were dichotomized to the lower 20% level.

### Statistical analysis

All participants were classified into quartile groups according to FPG variability values in the baseline characteristics. The data are expressed as mean ± standard deviation for continuous variables or number of participants (percentages) for categorical variables. The differences between groups of quartiles were calculated using ANOVA or χ^2^-test, as appropriate. The probability of PAD by quartiles of FPG variability was analyzed using the Kaplan-Meier method, and the log-rank test was used to determine the differences across the groups. We analyzed hazard ratios (HRs) and 95% CI for the development of PAD using Cox proportional hazards models for quartile groups of FPG variability. It was adjusted for age, sex, BMI, smoking status, alcohol consumption, regular exercise, income, hypertension medication, dyslipidemia medication, systolic blood pressure, total cholesterol, history of stroke, history of CAD, history of CKD, and mean FPG. Data were analyzed by an experienced expert statistician (S.Y. Hwang) using SAS Enterprise Guide 7.1 (SAS Institute Inc., Cary, NC, USA); A P-value < 0.05 was assumed to indicate statistical significance.

## Results

### Baseline characteristics of the study population

Table 1 shows the baseline characteristics of the study participants according to the CV quartiles for FPG variability. Among the 152,931 individuals with complete follow-up data, 16,863 (11.0%) had incident PAD during the median follow-up period of 8.3 years. The participants in the higher FPG-CV quartile were older and more likely to be of male sex than those in the lower quartile. Moreover, the groups with higher FPG variability had comorbidities, including an increased prevalence of IFG, dyslipidemia, and hypertension with elevated blood pressure level. According to the higher quartiles of FPG variability, participants had higher risk factors based on their unhealthy lifestyles, including smoking, alcohol consumption, and lack of regular exercise. However, FPG variability was not associated with BMI, total cholesterol level, or a history of stroke, CKD, CAD, or CHF. Similar associations of baseline characteristics among the study participants were described according to FPG variability quartiles as determined by SD (Additional file 1) and VIM (Additional file 2).

**Table 1:** Baseline characteristics of the participants according to the fasting plasma glucose variability (coefficient of variation)

	Q1	Q2	Q3	Q4	*P* value
CV range (%)	4.32 ± 1.47	8.02 ± 0.93	11.53 ± 1.16	19.61 ± 8.74	< 0.001
N	38,246	38,219	38,231	38,235	
Age (years)	55.83 ± 8.77	54.93 ± 8.34	55.08 ± 8.46	56.13 ± 8.95	< 0.001
Sex (male) (n, %)	19,959 (52.19)	22,325 (58.41)	23,421 (61.26)	24,928 (65.2)	< 0.0001
Body mass index (kg/m^2^)	23.82 ± 2.76	23.85 ± 2.81	23.85 ± 2.81	23.87 ± 2.89	0.208
Systolic BP (mmHg)	124.35 ± 15.48	124.55 ± 15.48	125.08 ± 15.48	126.6 ± 15.84	< 0.001
Diastolic BP (mmHg)	77.44 ± 10.1	77.83 ± 10.13	78.27 ± 10.2	79.04 ± 10.24	< 0.001
AST (IU/L)	25.39 ± 14.34	25.67 ± 14.08	26 ± 13.53	26.93 ± 16.7	< 0.001
ALT (IU/L)	24.08 ± 18.59	24.58 ± 18.82	24.82 ± 18.29	25.46 ± 19.47	< 0.001
GGT (IU/L)	33.45 ± 41.02	35.88 ± 44.34	37.61 ± 46.57	41.42 ± 54.19	< 0.001
Total cholesterol (mg/dL)	198.34 ± 35.37	198.26 ± 35.74	198.5 ± 35.89	198.8 ± 36.71	0.166
Mean FPG (mmol/L)	5.11 ± 0.51	5.08 ± 0.50	5.08 ± 0.49	5.31 ± 0.84	< 0.001
Smoking status (n, %)					< 0.001
Non-smoker	26,256 (68.65)	25,178 (65.88)	24,528 (64.16)	23,518 (61.51)	
Ex-smoker	3497 (9.14)	3525 (9.22)	3439 (9)	3209 (8.39)	
Current smoker	5574 (14.57)	6591 (17.25)	7250 (18.96)	8658 (22.64)	
Unknown	2919 (7.63)	2925 (7.65)	3014 (7.88)	2850 (7.45)	
Alcohol consumption (n, %)					< 0.001
Non-drinker	27,831 (72.77)	26,956 (70.53)	26,367 (68.97)	25,799 (67.47)	
Drinker	9295 (24.3)	10,277 (26.89)	10,892 (28.49)	11,640 (30.44)	
Unknown	1,120 (2.93)	986 (2.58)	972 (2.54)	796 (2.08)	
Regular exercise (n, %)					< 0.001
None	16,582 (43.36)	16,787 (43.92)	17,246 (45.11)	18,412 (48.15)	
Regular exercise	20,531 (53.68)	20,366 (53.29)	20,015 (52.35)	18,983 (49.65)	
Unknown	1133 (2.96)	1066 (2.79)	970 (2.54)	840 (2.2)	
Income (lower 20%)	4738 (12.39)	5130 (13.42)	5577 (14.59)	6387 (16.7)	< 0.001
IFG (%)	8465 (22.13)	9249 (24.2)	10,958 (28.66)	14,758 (38.6)	< 0.001
Hypertension	13,977 (36.54)	13,829 (36.18)	14,216 (37.18)	15,796 (41.31)	< 0.001
Dyslipidemia	7707 (20.15)	7551 (19.76)	7643 (19.99)	7933 (20.75)	0.005
History of stroke	200 (0.52)	173 (0.45)	187 (0.49)	215 (0.56)	0.172
History of chronic kidney disease	135 (0.35)	101 (0.26)	121 (0.32)	119 (0.31)	0.179
History of coronary artery disease	396 (1.04)	403 (1.05)	356 (0.93)	433 (1.13)	0.053
History of congestive heart failure	38 (0.1)	37 (0.1)	41 (0.11)	57 (0.15)	0.110
Use of anti-hypertension medication	14,441 (37.76)	14,002 (36.64)	14,023 (36.68)	15,298 (40.01)	< 0.001
Use of anti-dyslipidemia agent	4968 (12.99)	4774 (12.49)	4688 (12.26)	5053 (13.22)	< 0.001

P-value using ANOVA and Chi-square tests

Data are expressed as mean ± SD, or n (%)

CV: coefficient of variation; BP: blood pressure; AST: aspartate aminotransferase; ALT: alanine aminotransferase; GGT: γ-glutamyl transferase; FPG: fasting plasma glucose; IFG: impaired fasting glucose

### Effect of FPG variability on incident PAD

**Fig. 2:**
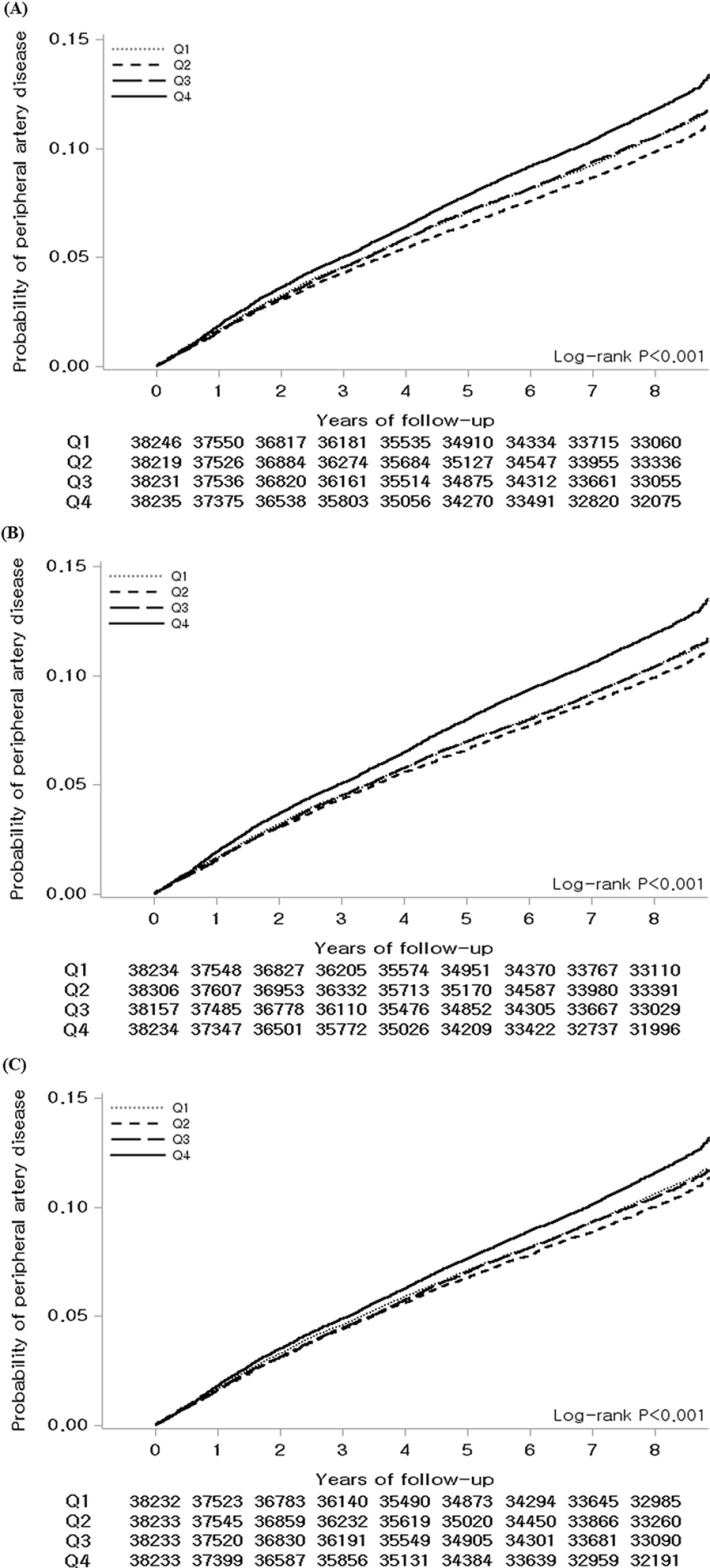
Kaplan–Meier estimates of the probability of peripheral artery disease expressed as quartiles of fasting plasma glucose (FPG) variability. **A** FPG variability (FPG–CV: coefficient of variance). **B** FPG variability (FPG–SD: standard deviation). **C** FPG variability (FPG–VIM: variability independent of the mean). P-value is from the log-rank test

Figure 2 presents the Kaplan–Meier curves of the probability of newly developing PAD for 8 years according to FPG-CV, FPG-SD, and FPG-VIM values. Higher levels of FPG variability were associated with an increased and progressive risk of incident PAD (all of log rank *P* < 0.001). Moreover, it was observed that the increased risk of developing PAD in the highest quartile group persisted compared to the lowest quartile group.

Table 2 describes the association between FPG variability, using CV, SD, and VIM values, and the risk of developing PAD. In all models, a higher risk of developing PAD was observed in the higher variability quartile groups than in the lowest quartile group. The results were consistent after stepwise adjustment for various confounding factors, including mean FPG. The adjusted HRs of prevalence of PAD in the highest quartile of the FPG variability group, as measured using CV, SD, and VIM, were 1.11 (95% CI 1.07–1.16), 1.11 (95% CI 1.06–1.16), and 1.11 (95% CI 1.07–1.16), respectively, compared to the lowest quartile group (all of *P* for trend < 0.001).

**Table 2:** Hazard ratios and 95% confidence intervals (CIs) of PAD by quartiles of FPG variability (CV, SD, and VIM)

	N	Events (n)	Follow-up duration (person-years)	Hazard ratio (95% CI)				
				Unadjusted	Model 1	Model 2	Model 3	Model 4
FPG variability (CV)								
Q1	38,246	4162	298,563	1	1	1	1	1
Q2	38,219	3898	299,318	0.94 (0.90, 0.98)	1.01 (0.96, 1.05)	1.00 (0.96, 1.05)	1.00 (0.95, 1.04)	1.00 (0.95, 1.04)
Q3	38,231	4171	297,885	1.01 (0.96, 1.05)	1.08 (1.04, 1.13)	1.07 (1.03, 1.12)	1.07 (1.02, 1.12)	1.07 (1.02, 1.12)
Q4	38,235	4632	293,916	1.13 (1.08, 1.18)	1.15 (1.11, 1.20)	1.14 (1.09, 1.19)	1.12 (1.07, 1.17)	1.11 (1.07, 1.16)
*P* for trend				< 0.001	< 0.001	< 0.001	< 0.001	< 0.001
FPG variability (SD)								
Q1	38,234	4119	298,809	1	1	1	1	1
Q2	38,306	3944	299,810	0.96 (0.91, 1.00)	1.02 (0.98, 1.07)	1.02 (0.97, 1.06)	1.01 (0.97, 1.06)	1.01 (0.97, 1.06)
Q3	38,157	4119	297,664	1.00 (0.96, 1.05)	1.08 (1.03, 1.13)	1.07 (1.02, 1.11)	1.05 (1.01, 1.10)	1.05 (1.01, 1.10)
Q4	38,234	4681	293,400	1.16 (1.11, 1.21)	1.18 (1.13, 1.23)	1.15 (1.11, 1.20)	1.12 (1.07, 1.17)	1.11 (1.06, 1.16)
*P* for trend				< 0.001	< 0.001	< 0.001	< 0.001	< 0.001
FPG variability (VIM)								
Q1	38,232	4196	298,180	1	1	1	1	1
Q2	38,233	3970	298,826	0.95 (0.91, 0.99)	1.01 (0.97, 1.06)	1.01 (0.97, 1.05)	1.00 (0.96, 1.05)	1.01 (0.96, 1.05)
Q3	38,233	4132	298,022	0.99 (0.94, 1.03)	1.07 (1.02, 1.12)	1.07 (1.02, 1.11)	1.07 (1.02, 1.11)	1.07 (1.03, 1.12)
Q4	38,233	4565	294,654	1.10 (1.06, 1.15)	1.13 (1.08, 1.18)	1.13 (1.08, 1.17)	1.11 (1.07, 1.16)	1.11 (1.07, 1.16)
*P* for trend				< 0.001	< 0.001	< 0.001	< 0.001	< 0.001

Model 1: Adjusted for age and sex

Model 2: Model 1+ body mass index, smoking status, alcohol consumption, regular exercise, and income

Model 3: Model 2+ antihypertensive medications, dyslipidemia medications, systolic blood pressure, total cholesterol, history of stroke, history of coronary artery disease, and history of chronic kidney disease

Model 4: Model 3 + mean FPG

PAD: peripheral artery disease; FPG: fasting plasma glucose; CV: coefficient of variation; SD: standard deviation; VIM: variability independent of the mean

In the sensitivity analysis, the association between FPG variability and incident PAD persisted even after the participants were excluded based on previously diagnosed diseases, including stroke (Additional file 3), CAD (Additional file 4), CHF (Additional file 5), CKD (Additional file 6), and current smokers (Additional file 7), or drinkers (Additional file 8), before and after adjusting for multi-variables and mean FPG.

**Fig. 3:**
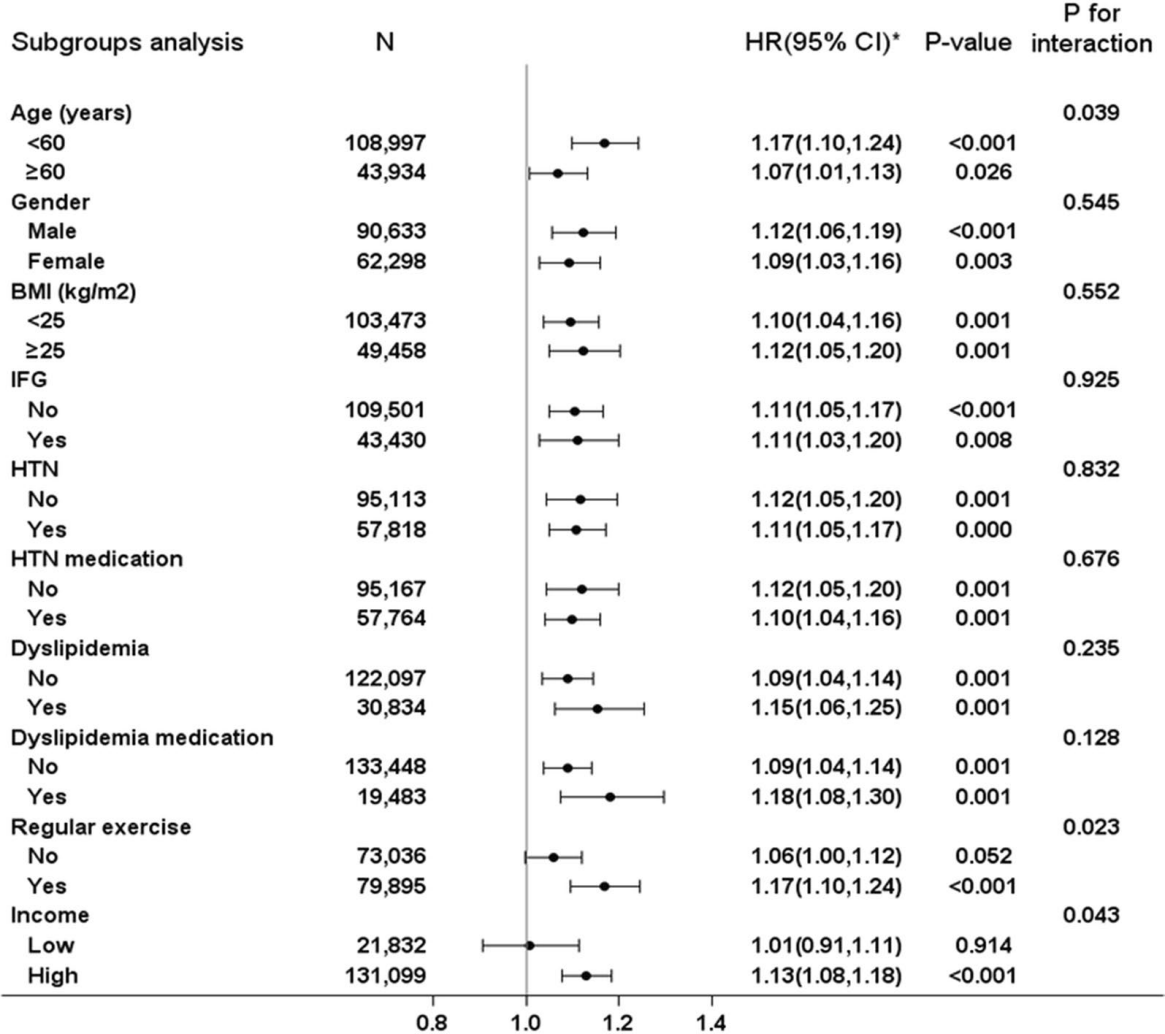
Association between fasting plasma glucose (FPG) variability and peripheral artery disease (PAD) in a subgroup analysis. *Hazard ratios for PAD in the highest quartile of FPG variability in reference to the lowest quartile using Cox proportional hazards regression models. BMI: body mass index; IFG: impaired fasting glucose; HTN: hypertension

In the stratification of subgroups by age, sex, BMI, IFG, hypertension, dyslipidemia, regular exercise, and income, p-values for interaction were significant in the subgroups of age (< 60 vs. ≥ 60 years), regular exercise (no vs. yes), and income (lower vs. higher) (Fig. 3). In the subgroups of < 60 years of age, regular exercisers, and those with higher income, the HRs of incident PAD were greater than for those > 60 years of age, non-exercisers, and those with lower incomes in the highest quartile of the FPG variability than in the lowest quartile group. The association between FPG variability and PAD was significant in most subgroups. However, in the no regular exercise subgroup and lower income subgroup, the risks of development PAD according to glycemic fluctuation were attenuated.

## Discussion

As proven in previous studies, glucose levels play an important role in developing atherosclerosis in population without diabetes. In the Atherosclerosis Risk in Communities (ARIC) study, HbA1c was significantly associated with the risk of CVD and all-cause mortality in adults without diabetes [22]. In nondiabetic population, elevated glucose level was also associated with increased risk of CAD [23]. Furthermore, post-stroke or post-CAD stress-induced hyperglycemia in patients without diabetes was associated with adverse outcomes including restenosis, diabetes or mortality [24–26]. Using a meta-analysis of 102 prospective studies, Sarwar et al. showed that the FPG levels were associated with atherosclerosis-related vascular disease in participants without diabetes [27]. A meta-analysis of prospective cohort studies with nondiabetic patients showed that glucose levels, regardless of postprandial glucose level or FPG level, were positively associated with CVD risk [28]. Similarly, a meta-analysis of 17 prospective cohort studies in the Asia Pacific region indicated that the FPG levels was a critical risk factor for CVD, and that there were significant potential benefits to reducing fasting glucose levels to at least 4.9 mmol/L in participants with/without diabetes [29]. Overall, hyperglycemia, even in the nondiabetic range, seems to have an adverse effect on cardiometabolic diseases, as confirmed in diabetes patients.

Additionally, glycemic variability has been suggested as a novel predictor of cardiovascular complications and reducing it is an emerging glycemic target. Several observational studies [9] and post-hoc analysis of trials, including the *Action in Diabetes and Vascular Disease: Preterax and Diamicron MR Controlled Evaluation* (ADVANCE) trial [11], the Veterans Affairs Diabetes Trial (VADT) [30], the Trial Comparing Cardiovascular Safety of Insulin Degludec vs. Insulin Glargine in Patients with Type 2 Diabetes at High Risk of Cardiovascular Events (DEVOTE) [31], the Action to Control Cardiovascular Risk in Diabetes (ACCORD) trial [32], and the Empagliflozin Cardiovascular Outcome Event (EMPA-REG OUTCOME) trial [33] demonstrated a positive association between visit-to-visit glycemic variability and the risk of micro/macrovascular disease in participants with type 2 diabetes. Furthermore, long-term glycemic variability has also been suggested to affect cardiovascular disease and mortality even in individuals without diabetes [18, 34].

Recently, several studies have investigated the effect of glycemic variability on PAD development in patients with diabetes. In an Italian multicenter study, Penno et al. showed that HbA1c-SD correlated with lower extremity PAD and severe PAD, such as ulceration/gangrene in patients with type 2 diabetes [35]. A meta-analysis showed that increased HbA1c variability was associated with a higher risk of severe PAD in patients with type 2 diabetes [9]. In a population-based cohort in Taiwan, Yang et al. demonstrated a significant association between FPG-CV and development of PAD, independent of HbA1c levels, in participants with type 2 diabetes [36]. However, many confounding factors might affect their relationship, such as compliance of anti-diabetic medication, duration of diabetes, and anti-atherosclerotic effects of medications. Recently, Sun et al. showed a positive relationship between FPG variability and the risk of PAD even in individuals without diabetes, using the ARIC study cohort from US database [37]. However, Sun et al.’s study was performed based on only three measurements of FPG levels for the calculation of glucose variability. Furthermore, they simply adjusted for baseline FPG as a confounding factor along with other cardiovascular risk factors and did not adjust the mean FPG levels. Because FPG is known as a critical risk factor for atherosclerotic CVD, in addition to glycemic variability, the mean FPG levels during study period might also affect the final result. In addition, the present study, that included an Asian population with a relatively lower risk of PAD than the Caucasian population, may broaden the insights into the relationship between glycemic variability and atherosclerosis.

The pathophysiological mechanisms that link the association between long-term FPG variability and PAD are still unknown; thus, further research is required. There are some possible explanations for why high glycemic variability could be a risk factor for PAD incidence. First, glycemic variability, regardless of persistent hyperglycemia, could play a crucial role in atherosclerosis by increasing chronic inflammation, oxidative stress, endothelial dysfunction, and insulin resistance [12–15]. Second, glycemic variation may lead to pancreatic β-cell dysfunction and apoptosis [38]. Furthermore, metabolic memory and chromatin remodeling caused by repeated glycemic fluctuations, could induce atherosclerosis [39]. Third, glycemic variability may induce cardiovascular autonomic dysfunction with sympathetic activation that may contribute to atherosclerosis [40–42]. It has been reported that patients with PAD have decreased heart rate variability than those without PAD in type 2 diabetes, which implies cardiovascular autonomic dysfunction [40]. Lastly, individuals with high glycemic variability tended to have more traditional risk factors, such as advanced age, high BMI, smoking history, high blood pressure, and dyslipidemia. However, the present study tried to alleviate their influence using various sensitivity analyses and extensive adjustment for diverse confounding factors.

Different effects of FPG variability on the risk of PAD were observed across subgroups of age, exercise, and income. In younger individuals (< 60 years), regular exercisers, and those with higher income, FPG variability was associated with a higher risk of PAD than in those of older age (≥ 60 years), non-regular exercisers, and those with lower income. Although statistically insignificant, Sun et al. also showed that individuals aged < 60 years had a higher risk of PAD using FPG-CV and FPG-SD than individuals aged ≥ 60 years among those without diabetes [37]. In epidemiologic studies, PAD is more common in older age [43], sedentary lifestyle [44], and low socioeconomic status [45, 46]. These results may suggest that increased FPG variability had a greater impact on the risk of PAD in individuals with relatively favorable conditions for cardiovascular risk.

This study has several limitations. First, a hereditary limitation of the cohort using national health examinations may contain a potential selection bias. It was probably made up of healthier and movable people or health-conscious people. Nevertheless, the NHIS-HEALS is a representative data of Korean population with high participation rates of the wide-ranging health examination program (up to 74.8% in 2014). Second, other assessments of glycemic levels, including HbA1c or oral glucose tolerance tests, were not used in the health checkup data by the NHIS. Third, the causal relationship between FPG variability and incident PAD could not be confirmed. In addition, PAD was defined by physicians’ diagnostic codes of the ICD-10; thus, under- or over-diagnosis of PAD could be possible. In this study, the prevalence of PAD was 11%, similar to that (10.7%) of a large cohort study of individuals aged 40 or older in the United States [3]. Previous studies also defined PAD based on medical claim codes [3, 36]. We used the ICD-10 code number according to the American Heart Association [21]. Lastly, although we tried to adjust for diverse confounding variables that likely influenced the incidence of PAD, the probability of residual confounding variables cannot be excluded. However, this study has several noteworthy strengths. This study had a large sample size, extensive information about potential confounding variables, and sufficient duration of observation, using a standardized database validated by the Korean government. In particular, we showed coincident and consistent implications for PAD incidence according to long-term glycemic variability determined by three different indices, FPG-CV, FPG-SD, and FPG-VIM, even after adjusting for multiple confounding variables including average FPG. Additionally, we used samples taken from the participants representing the entire Korean population; thus, these findings may reflect the real-world situation.

## Conclusions

In conclusion, regardless of average FPG levels and other risk factors, visit-to-visit FPG variability was positively associated with long-term risk of PAD in a general population without diabetes. These findings revealed that reducing glycemic variability may be a potential target for the prevention of PAD. Further investigation is needed to generalize these findings and to better understand the development of PAD, especially in the population without diabetes.

## Supplementary Information

**Additional file 1.** Baseline characteristics of the participants according to the fasting plasma glucose variability (standard deviation).

**Additional file 2.** Baseline characteristics of the participants according to the fasting plasma glucose variability (variability independent of the mean).

**Additional file 3.** Hazard ratios and 95% confidence intervals (CIs) for PAD according to quartiles of FPG variability (CV, SD, and VIM) in participants without stroke.

**Additional file 4.** Hazard ratios and 95% confidence intervals (CIs) for PAD according to quartiles of FPG variability (CV, SD, and VIM) in participants without coronary artery disease.

**Additional file 5.** Hazard ratios and 95% confidence intervals (CIs) of PAD by quartiles of FPG variability (CV, SD, and VIM) in participants without congestive heart failure.

**Additional file 6.** Hazard ratios and 95% confidence intervals (CIs) of PAD by quartiles of FPG variability (CV, SD, and VIM) in participants without chronic kidney disease.

**Additional file 7.** Hazard ratios and 95% confidence intervals (CIs) of PAD by quartiles of FPG variability (CV, SD, and VIM) in participants without a history of current smoker.

**Additional file 8.** Hazard ratios and 95% confidence intervals (CIs) of PAD by quartiles of FPG variability (CV, SD, and VIM) in the study sample after excluding alcohol consumers.

## Data Availability

The datasets generated and/or analyzed during the current study are not publicly available but are available from the corresponding author on reasonable request.

## Abbreviations

AHA: American Heart Association
BMI: Body mass index
CAD: Coronary artery disease
CI: Confidence interval
CHF: Congestive heart failure
CKD: Chronic kidney disease
CV: Coefficient of variation
CVD: Cardiovascular disease
FPG: Fasting plasma glucose
HbA1c: Hemoglobin A1C
HRs: Hazard ratios
ICD-10: International Classification of Diseases Tenth Revision
IFG: Impaired fasting glucose
NHIS-HEALS: National Health Insurance Service-National Health Screening Cohort
PAD: Peripheral artery disease
SD: Standard deviation
VIM: Variability independent of the mean
