# Supplementary material for: Implications of fasting plasma glucose variability on the risk of incident peripheral artery disease in a population without diabetes: a nationwide population-based cohort study
Source: Cardiovasc Diabetol. 2022 Jan 31;21:15. doi: 10.1186/s12933-022-01448-1 (PMC8805289; doi:10.1186/s12933-022-01448-1)
Supplement: Supplementary file 1 — Additional file 1. Baseline characteristics of the participants according to the fasting plasma glucose variability (standard deviation). [file 12933_2022_1448_MOESM1_ESM.docx]

Additional file 1. Baseline characteristics of the participants according to the fasting plasma glucose variability (standard deviation)

|  | Q1 | Q2 | Q3 | Q4 |
| --- | --- | --- | --- | --- |
| N | 38,234 | 38,306 | 38,157 | 38,234 |
| Age (years) | 55.71±8.74 | 54.94±8.36 | 55.08±8.46 | 56.24±8.96 |
| Sex (male) (n, %) | 19,425(50.81) | 22,216(58) | 23,644(61.97) | 25,348(66.3) |
| Body mass index (kg/m2) | 23.76±2.75 | 23.81±2.81 | 23.88±2.8 | 23.94±2.91 |
| Systolic BP (mmHg) | 123.94±15.46 | 124.37±15.4 | 125.24±15.45 | 127.04±15.89 |
| Diastolic BP (mmHg) | 77.21±10.08 | 77.74±10.14 | 78.37±10.14 | 79.25±10.26 |
| AST (IU/L) | 25.26±14.57 | 25.57±13.55 | 26.06±13.6 | 27.11±16.86 |
| ALT (IU/L) | 23.76±18.93 | 24.41±18.1 | 24.95±18.34 | 25.84±19.74 |
| GGT (IU/L) | 32.38±39.47 | 35.25±42.92 | 38.08±47.77 | 42.65±55.23 |
| Total cholesterol (mg/dL) | 197.83±35.34 | 198.21±35.56 | 198.66±35.93 | 199.2±36.87 |
| Mean FPG (mmol/L) | 5.02±0.49 | 5.04±0.48 | 5.10±0.47 | 5.42±0.82 |
| Smoking status (n, %) |  |  |  |  |
| Non-smoker | 26,469(69.23) | 25,343(66.16) | 24,303(63.69) | 23,365(61.11) |
| Ex-smoker | 3,324(8.69) | 3,564(9.3) | 3,479(9.12) | 3,303(8.64) |
| Current smoker | 5,519(14.43) | 6,511(17) | 7,381(19.34) | 8,662(22.66) |
| Unknown | 2,922(7.64) | 2,888(7.54) | 2,994(7.85) | 2,904(7.6) |
| Alcohol consumption (n, %) |  |  |  |  |
| Non-drinker | 28,192(73.74) | 27,160(70.9) | 26,191(68.64) | 25,410(66.46) |
| Drinker | 8,889(23.25) | 10,180(26.58) | 11,028(28.9) | 12,007(31.4) |
| Unknown | 1,153(3.02) | 966(2.52) | 938(2.46) | 817(2.14) |
| Regular exercise (n, %) |  |  |  |  |
| None | 16,730(43.76) | 16,922(44.18) | 17,077(44.75) | 18,298(47.86) |
| Regular exercise | 20,342(53.2) | 20,349(53.12) | 20,130(52.76) | 19,074(49.89) |
| Unknown | 1,162(3.04) | 1,035(2.7) | 950(2.49) | 862(2.25) |
| Income (lower 20%) | 4,886(12.78) | 5,111(13.34) | 5,478(14.36) | 6,357(16.63) |
| IFG (%) | 6,558(17.15) | 8,470(22.11) | 11,663(30.57) | 16,739(43.78) |
| Hypertension | 13,561(35.47) | 13,651(35.64) | 14,289(37.45) | 16,317(42.68) |
| Dyslipidemia | 7,477(19.56) | 7,502(19.58) | 7,687(20.15) | 8,168(21.36) |
| History of Stroke | 192(0.5) | 185(0.48) | 180(0.47) | 218(0.57) |
| History of chronic kidney disease | 132(0.35) | 104(0.27) | 120(0.31) | 120(0.31) |
| History of coronary artery disease | 394(1.03) | 376(0.98) | 386(1.01) | 432(1.13) |
| History of congestive heart failure | 39(0.1) | 39(0.1) | 38(0.1) | 57(0.15) |
| Use of anti-hypertension medication | 14,167(37.05) | 13,875(36.22) | 14,091(36.93) | 15,631(40.88) |
| Use of anti-dyslipidemia agent | 4,808(12.58) | 4,680(12.22) | 4,756(12.46) | 5,239(13.7) |

P-value using ANOVA and Chi-square tests

Data are expressed as mean ± SD, or n (%).

BP, blood pressure; AST, aspartate aminotransferase; ALT, alanine aminotransferase; GGT, γ-glutamyl transferase; FPG, fasting plasma glucose; IFG, impaired fasting glucose
