# Supplementary material for: Implications of fasting plasma glucose variability on the risk of incident peripheral artery disease in a population without diabetes: a nationwide population-based cohort study
Source: Cardiovasc Diabetol. 2022 Jan 31;21:15. doi: 10.1186/s12933-022-01448-1 (PMC8805289; doi:10.1186/s12933-022-01448-1)
Supplement: Supplementary file 2 — Additional file 2. Baseline characteristics of the participants according to the fasting plasma glucose variability (variability independent of the mean). [file 12933_2022_1448_MOESM2_ESM.docx]

Additional file 2. Baseline characteristics of the participants according to the fasting plasma glucose variability (variability independent of the mean)

|  | Q1 | Q2 | Q3 | Q4 |
| --- | --- | --- | --- | --- |
| N | 38,232 | 38,233 | 38,233 | 38,233 |
| Age (years) | 55.91±8.79 | 55.06±8.41 | 55±8.41 | 55.99±8.93 |
| Sex (male) (n, %) | 20,486(53.58) | 22,390(58.56) | 23,345(61.06) | 24,412(63.85) |
| Body mass index (kg/m2) | 23.89±2.76 | 23.87±2.82 | 23.83±2.81 | 23.79±2.88 |
| Systolic BP (mmHg) | 124.76±15.52 | 124.8±15.59 | 124.95±15.45 | 126.08±15.78 |
| Diastolic BP (mmHg) | 77.67±10.1 | 77.93±10.16 | 78.22±10.22 | 78.75±10.23 |
| AST (IU/L) | 25.54±14.82 | 25.67±13.34 | 26.02±13.93 | 26.76±16.57 |
| ALT (IU/L) | 24.39±18.56 | 24.58±18.27 | 24.78±18.78 | 25.19±19.58 |
| GGT (IU/L) | 34.46±42.78 | 36.18±44.07 | 37.64±47.62 | 40.09±52.25 |
| Total cholesterol (mg/dL) | 198.82±35.59 | 198.38±35.68 | 198.31±35.9 | 198.39±36.55 |
| Mean FPG (mmol/L) | 5.20±0.52 | 5.12±0.50 | 5.07±0.50 | 5.20±0.83 |
| Smoking status (n, %) |  |  |  |  |
| Non-smoker | 25,938(67.84) | 25,221(65.97) | 24,576(64.28) | 23,745(62.11) |
| Ex-smoker | 3,665(9.59) | 3,496(9.14) | 3,366(8.8) | 3,143(8.22) |
| Current smoker | 5,684(14.87) | 6,615(17.3) | 7,258(18.98) | 8,516(22.27) |
| Unknown | 2,945(7.7) | 2,901(7.59) | 3,033(7.93) | 2,829(7.4) |
| Alcohol consumption (n, %) |  |  |  |  |
| Non-drinker | 27,448(71.79) | 26,888(70.33) | 26,446(69.17) | 26,171(68.45) |
| Drinker | 9,671(25.3) | 10,373(27.13) | 10,808(28.27) | 11,252(29.43) |
| Unknown | 1,113(2.91) | 972(2.54) | 979(2.56) | 810(2.12) |
| Regular exercise (n, %) |  |  |  |  |
| None | 16,381(42.85) | 16,862(44.1) | 17,331(45.33) | 18,453(48.26) |
| Regular exercise | 20,732(54.23) | 20,319(53.15) | 19,926(52.12) | 18,918(49.48) |
| Unknown | 1,119(2.93) | 1,052(2.75) | 976(2.55) | 862(2.25) |
| Income (lower 20%) | 4,692(12.27) | 5,138(13.44) | 5,639(14.75) | 6,363(16.64) |
| IFG (%) | 10,321(27) | 9,878(25.84) | 10,406(27.22) | 12,825(33.54) |
| Hypertension | 14,399(37.66) | 14,117(36.92) | 14,080(36.83) | 15,222(39.81) |
| Dyslipidaemia | 7,910(20.69) | 7,612(19.91) | 7,586(19.84) | 7,726(20.21) |
| history of Stroke | 195(0.51) | 189(0.49) | 184(0.48) | 207(0.54) |
| history of chronic kidney disease | 134(0.35) | 104(0.27) | 115(0.3) | 123(0.32) |
| history of coronary artery disease | 409(1.07) | 400(1.05) | 370(0.97) | 409(1.07) |
| history of congestive heart failure | 41(0.11) | 26(0.07) | 51(0.13) | 55(0.14) |
| use of anti hypertension medication | 14,675(38.38) | 14,215(37.18) | 13,961(36.52) | 14,913(39.01) |
| use of anti dyslipidemia agent | 5,111(13.37) | 4,811(12.58) | 4,693(12.27) | 4,868(12.73) |

P-value using ANOVA and Chi-square tests

Data are expressed as mean ± SD, or n (%).

BP, blood pressure; AST, aspartate aminotransferase; ALT, alanine aminotransferase; GGT, γ-glutamyl transferase; FPG, fasting plasma glucose; IFG, impaired fasting glucose
