# Supplementary material for: Implications of fasting plasma glucose variability on the risk of incident peripheral artery disease in a population without diabetes: a nationwide population-based cohort study
Source: Cardiovasc Diabetol. 2022 Jan 31;21:15. doi: 10.1186/s12933-022-01448-1 (PMC8805289; doi:10.1186/s12933-022-01448-1)
Supplement: Supplementary file 4 — Additional file 4. Hazard ratios and 95% confidence intervals (CIs) for PAD according to quartiles of FPG variability (CV, SD, and VIM) in participants without coronary artery disease. [file 12933_2022_1448_MOESM4_ESM.docx]

Additional file 4. Hazard ratios and 95% confidence intervals (CIs) for PAD according to quartiles of FPG variability (CV, SD, and VIM) in participants without coronary artery disease

|  | N | Events (n) | Follow-up duration (person-years) | Hazard Ratio (95% CI) | | | | |
| --- | --- | --- | --- | --- | --- | --- | --- | --- |
|  |  |  |  | Unadjusted | Model 1 | Model 2 | Model 3 | Model 4 |
| FPG variability (CV) | |  |  |  |  |  |  |  |
| Q1 | 37,850 | 4,105 | 295,640 | 1 | 1 | 1 | 1 | 1 |
| Q2 | 3,7821 | 3,855 | 296,302 | 0.94 (0.90,0.98) | 1.01 (0.97,1.06) | 1.00 (0.96,1.05) | 1.00 (0.96,1.04) | 1.00 (0.96,1.04) |
| Q3 | 37,837 | 4,120 | 294,925 | 1.01 (0.96,1.05) | 1.08 (1.04,1.13) | 1.07 (1.03,1.12) | 1.07 (1.02,1.12) | 1.07 (1.02,1.12) |
| Q4 | 37,835 | 4,586 | 290,879 | 1.14 (1.09,1.18) | 1.16 (1.11,1.21) | 1.15 (1.10,1.20) | 1.12 (1.07,1.17) | 1.12 (1.07,1.17) |
| *P* for trend | |  |  | <0.001 | <0.001 | <0.001 | <0.001 | <0.001 |
| FPG variability (SD) | |  |  |  |  |  |  |  |
| Q1 | 37,840 | 4,066 | 295,879 | 1 | 1 | 1 | 1 | 1 |
| Q2 | 37,930 | 3,899 | 296,989 | 0.96 (0.92,1.00) | 1.02 (0.98,1.07) | 1.02 (0.97,1.06) | 1.01 (0.97,1.06) | 1.01 (0.97,1.06) |
| Q3 | 37,734 | 4,068 | 294,474 | 1.01 (0.96,1.05) | 1.08 (1.03,1.13) | 1.07 (1.02,1.11) | 1.05 (1.01,1.10) | 1.05 (1.01,1.10) |
| Q4 | 37,839 | 4,633 | 290,404 | 1.16 (1.11,1.21) | 1.18 (1.13,1.23) | 1.16 (1.11,1.21) | 1.12 (1.07,1.17) | 1.11 (1.07,1.16) |
| *P* for trend | |  |  | <0.001 | <0.001 | <0.001 | <0.001 | <0.001 |
| FPG variability (VIM) | |  |  |  |  |  |  |  |
| Q1 | 37,835 | 4,140 | 295,232 | 1 | 1 | 1 | 1 | 1 |
| Q2 | 37,837 | 3,924 | 295,852 | 0.95 (0.91,0.99) | 1.01 (0.97,1.06) | 1.01 (0.97,1.05) | 1.01 (0.96,1.05) | 1.01 (0.97,1.05) |
| Q3 | 37,835 | 4,081 | 295,047 | 0.99 (0.95,1.03) | 1.07 (1.02,1.12) | 1.07 (1.02,1.11) | 1.07 (1.02,1.12) | 1.07 (1.03,1.12) |
| Q4 | 37,836 | 4,521 | 291,615 | 1.11 (1.06,1.15) | 1.13 (1.09,1.18) | 1.13 (1.08,1.18) | 1.12 (1.07,1.17) | 1.12 (1.07,1.17) |
| *P* for trend | |  |  | <0.001 | <0.001 | <0.001 | <0.001 | <0.001 |

Model 1: Adjusted for age and sex

Model 2: Model 1+ body mass index, smoking status, alcohol consumption, regular exercise, and income

Model 3: Model 2+ antihypertensive medication, dyslipidemia medication, systolic blood pressure, total cholesterol, history of stroke, and history of chronic kidney disease

Model 4: Model 3 + mean FPG

PAD, peripheral artery disease; FPG, fasting plasma glucose; CV, coefficient of variation; SD, standard deviation; VIM, variability independent of the mean
