# Supplementary material for: Implications of fasting plasma glucose variability on the risk of incident peripheral artery disease in a population without diabetes: a nationwide population-based cohort study
Source: Cardiovasc Diabetol. 2022 Jan 31;21:15. doi: 10.1186/s12933-022-01448-1 (PMC8805289; doi:10.1186/s12933-022-01448-1)
Supplement: Supplementary file 5 — Additional file 5. Hazard ratios and 95% confidence intervals (CIs) of PAD by quartiles of FPG variability (CV, SD, and VIM) in participants without congestive heart failure. [file 12933_2022_1448_MOESM5_ESM.docx]

Additional file 5. Hazard ratios and 95% confidence intervals (CIs) of PAD by quartiles of FPG variability (CV, SD, and VIM) in participants without congestive heart failure

|  | N | Events (n) | Follow-up duration (person-years) | Hazard Ratio (95% CI) | | | | |
| --- | --- | --- | --- | --- | --- | --- | --- | --- |
|  |  |  |  | Unadjusted | Model 1 | Model 2 | Model 3 | Model 4 |
| FPG variability (CV) | |  |  |  |  |  |  |  |
| Q1 | 38,172 | 4,153 | 298,029 | 1 | 1 | 1 | 1 | 1 |
| Q2 | 38,209 | 3,897 | 299,288 | 0.94 (0.90,0.98) | 1.01 (0.96,1.05) | 1.01 (0.96,1.05) | 1.00 (0.95,1.04) | 1.00 (0.95,1.04) |
| Q3 | 38,188 | 4,166 | 297,566 | 1.01 (0.96,1.05) | 1.08 (1.04,1.13) | 1.08 (1.04,1.13) | 1.07 (1.02,1.12) | 1.07 (1.02,1.12) |
| Q4 | 38,189 | 4,625 | 293,631 | 1.13 (1.08,1.18) | 1.15 (1.11,1.20) | 1.15 (1.11,1.20) | 1.12 (1.07,1.17) | 1.11 (1.07,1.16) |
| *P* for trend | |  |  | <0.001 | <0.001 | <0.001 | <0.001 | <0.001 |
| FPG variability (SD) | |  |  |  |  |  |  |  |
| Q1 | 38,195 | 4,116 | 298,545 | 1 | 1 | 1 | 1 | 1 |
| Q2 | 38,267 | 3,939 | 299,542 | 0.95 (0.91,1.00) | 1.02 (0.98,1.07) | 1.02 (0.97,1.06) | 1.01 (0.97,1.06) | 1.01 (0.97,1.06) |
| Q3 | 38,108 | 4,113 | 297,319 | 1.00 (0.96,1.05) | 1.08 (1.03,1.13) | 1.07 (1.02,1.11) | 1.05 (1.01,1.10) | 1.05 (1.01,1.10) |
| Q4 | 38,188 | 4,673 | 293,109 | 1.16 (1.11,1.21) | 1.18 (1.13,1.23) | 1.15 (1.11,1.20) | 1.12 (1.07,1.17) | 1.11 (1.06,1.16) |
| *P* for trend | |  |  | <0.001 | <0.001 | <0.001 | <0.001 | <0.001 |
| FPG variability (VIM) | |  |  |  |  |  |  |  |
| Q1 | 38,189 | 4,195 | 297,890 | 1 | 1 | 1 | 1 | 1 |
| Q2 | 38,191 | 3,959 | 298,544 | 0.94 (0.90,0.98) | 1.01 (0.97,1.05) | 1.01 (0.96,1.05) | 1.00 (0.96,1.05) | 1.00 (0.96,1.05) |
| Q3 | 38,189 | 4,126 | 297,717 | 0.99 (0.94,1.03) | 1.07 (1.02,1.12) | 1.06 (1.02,1.11) | 1.07 (1.02,1.11) | 1.07 (1.02,1.12) |
| Q4 | 38,189 | 4,561 | 294,363 | 1.10 (1.06,1.15) | 1.13 (1.08,1.18) | 1.13 (1.08,1.17) | 1.11 (1.07,1.16) | 1.11 (1.07,1.16) |
| *P* for trend | |  |  | <0.001 | <0.001 | <0.001 | <0.001 | <0.001 |

Model 1: Adjusted for age and sex

Model 2: Model 1+ body mass index, smoking status, alcohol consumption, regular exercise, and income

Model 3: Model 2+ antihypertensive medication, dyslipidemia medicationa, systolic blood pressure, total cholesterol, history of stroke, history of coronary artery disease, and history of chronic kidney disease

Model 4: Model 3 + mean FPG

PAD, peripheral artery disease; FPG, fasting plasma glucose; CV, coefficient of variation; SD, standard deviation; VIM, variability independent of the mean
