# Supplementary material for: Implications of fasting plasma glucose variability on the risk of incident peripheral artery disease in a population without diabetes: a nationwide population-based cohort study
Source: Cardiovasc Diabetol. 2022 Jan 31;21:15. doi: 10.1186/s12933-022-01448-1 (PMC8805289; doi:10.1186/s12933-022-01448-1)
Supplement: Supplementary file 7 — Additional file 7. Hazard ratios and 95% confidence intervals (CIs) of PAD by quartiles of FPG variability (CV, SD, and VIM) in participants without a history of current smoker. [file 12933_2022_1448_MOESM7_ESM.docx]

Additional file 7. Hazard ratios and 95% confidence intervals (CIs) of PAD by quartiles of FPG variability (CV, SD, and VIM) in participants without a history of current smoker

|  | N | Events (n) | Follow-up duration (person-years) | Hazard Ratio (95% CI) | | | | |
| --- | --- | --- | --- | --- | --- | --- | --- | --- |
|  |  |  |  | Unadjusted | Model 1 | Model 2 | Model 3 | Model 4 |
| FPG variability (CV) | |  |  |  |  |  |  |  |
| Q1 | 31,215 | 3,581 | 243,309 | 1 | 1 | 1 | 1 | 1 |
| Q2 | 31,212 | 3,374 | 243,863 | 0.94 (0.90,0.99) | 1.00 (0.96,1.05) | 1.00 (0.95,1.05) | 1.00 (0.95,1.04) | 1.00 (0.95,1.05) |
| Q3 | 31,217 | 3,584 | 243,009 | 1.00 (0.96,1.05) | 1.07 (1.02,1.12) | 1.05 (1.01,1.10) | 1.05 (1.00,1.10) | 1.05 (1.00,1.10) |
| Q4 | 31,214 | 4,068 | 239,096 | 1.16 (1.10,1.21) | 1.16 (1.11,1.21) | 1.14 (1.09,1.19) | 1.12 (1.07,1.17) | 1.12 (1.07,1.17) |
| *P* for trend | |  |  | <0.001 | <0.001 | <0.001 | <0.001 | <0.001 |
| FPG variability (SD) | |  |  |  |  |  |  |  |
| Q1 | 31,243 | 3,527 | 243,848 | 1 | 1 | 1 | 1 | 1 |
| Q2 | 31,201 | 3,385 | 243,930 | 0.96 (0.92,1.01) | 1.02 (0.97,1.07) | 1.01 (0.97,1.06) | 1.01 (0.97,1.06) | 1.01 (0.97,1.06) |
| Q3 | 31,166 | 3,581 | 242,521 | 1.02 (0.98,1.07) | 1.08 (1.04,1.14) | 1.07 (1.02,1.12) | 1.06 (1.01,1.11) | 1.06 (1.01,1.11) |
| Q4 | 31,248 | 4,114 | 238,976 | 1.19 (1.14,1.24) | 1.18 (1.13,1.24) | 1.16 (1.11,1.21) | 1.12 (1.08,1.18) | 1.12 (1.07,1.17) |
| *P* for trend | |  |  | <0.001 | <0.001 | <0.001 | <0.001 | <0.001 |
| FPG variability (VIM) | |  |  |  |  |  |  |  |
| Q1 | 31,215 | 3,618 | 242,984 | 1 | 1 | 1 | 1 | 1 |
| Q2 | 31,214 | 3,456 | 243,414 | 0.95 (0.91,1.00) | 1.01 (0.97,1.06) | 1.01 (0.96,1.06) | 1.01 (0.96,1.06) | 1.01 (0.96,1.06) |
| Q3 | 31,215 | 3,526 | 243,209 | 0.97 (0.93,1.02) | 1.05 (1.00,1.10) | 1.04 (0.99,1.09) | 1.04 (1.00,1.09) | 1.05 (1.00,1.10) |
| Q4 | 31,214 | 4,007 | 239,668 | 1.12 (1.07,1.17) | 1.13 (1.08,1.19) | 1.12 (1.07,1.18) | 1.11 (1.07,1.17) | 1.11 (1.07,1.17) |
| *P* for trend | |  |  | <0.001 | <0.001 | <0.001 | <0.001 | <0.001 |

Model 1: Adjusted for age and sex

Model 2: Model 1+ body mass index, alcohol consumption, regular exercise, and income

Model 3: Model 2+ antihypertensive medication, dyslipidemia medication, systolic blood pressure, total cholesterol, history of stroke, history of coronary artery disease, and history of chronic kidney disease

Model 4: Model 3 + mean FPG

PAD, peripheral artery disease; FPG, fasting plasma glucose; CV, coefficient of variation; SD, standard deviation; VIM, variability independent of the mean
