# Supplementary material for: Implications of fasting plasma glucose variability on the risk of incident peripheral artery disease in a population without diabetes: a nationwide population-based cohort study
Source: Cardiovasc Diabetol. 2022 Jan 31;21:15. doi: 10.1186/s12933-022-01448-1 (PMC8805289; doi:10.1186/s12933-022-01448-1)
Supplement: Supplementary file 8 — Additional file 8. Hazard ratios and 95% confidence intervals (CIs) of PAD by quartiles of FPG variability (CV, SD, and VIM) in the study sample after excluding alcohol consumers. [file 12933_2022_1448_MOESM8_ESM.docx]

Additional file 8. Hazard ratios and 95% confidence intervals (CIs) of PAD by quartiles of FPG variability (CV, SD, and VIM) in the study sample after excluding alcohol consumers

|  | N | Events (n) | Follow-up duration (person-years) | Hazard Ratio (95% CI) | | | | |
| --- | --- | --- | --- | --- | --- | --- | --- | --- |
|  |  |  |  | Unadjusted | Model 1 | Model 2 | Model 3 | Model 4 |
| FPG variability (CV) | |  |  |  |  |  |  |  |
| Q1 | 27,706 | 3,199 | 215,607 | 1 | 1 | 1 | 1 | 1 |
| Q2 | 27,700 | 2,996 | 216,229 | 0.93 (0.89,0.98) | 1.00 (0.95,1.05) | 1.00 (0.95,1.05) | 0.99 (0.94,1.04) | 0.99 (0.95,1.04) |
| Q3 | 27,718 | 3,214 | 215,352 | 1.01 (0.96,1.06) | 1.08 (1.03,1.13) | 1.07 (1.02,1.13) | 1.07 (1.02,1.12) | 1.07 (1.02,1.12) |
| Q4 | 27,703 | 3,573 | 211,916 | 1.14 (1.08,1.19) | 1.15 (1.10,1.21) | 1.13 (1.08,1.19) | 1.11 (1.06,1.17) | 1.11 (1.06,1.16) |
| *P* for trend | |  |  | <0.001 | <0.001 | <0.001 | <0.001 | <0.001 |
| FPG variability (SD) | |  |  |  |  |  |  |  |
| Q1 | 27,710 | 3,158 | 215,891 | 1 | 1 | 1 | 1 | 1 |
| Q2 | 27,644 | 3,005 | 215,871 | 0.95 (0.91,1.00) | 1.02 (0.97,1.07) | 1.02 (0.97,1.07) | 1.01 (0.96,1.06) | 1.01 (0.96,1.06) |
| Q3 | 27,745 | 3,185 | 215,573 | 1.01 (0.96,1.06) | 1.08 (1.03,1.13) | 1.07 (1.01,1.12) | 1.05 (1.00,1.11) | 1.05 (1.00,1.11) |
| Q4 | 27,728 | 3,634 | 211,767 | 1.17 (1.12,1.23) | 1.18 (1.12,1.23) | 1.15 (1.10,1.21) | 1.12 (1.07,1.18) | 1.12 (1.06,1.17) |
| *P* for trend | |  |  | <0.001 | <0.001 | <0.001 | <0.001 | <0.001 |
| FPG variability (VIM) | |  |  |  |  |  |  |  |
| Q1 | 27,706 | 3,229 | 215,377 | 1 | 1 | 1 | 1 | 1 |
| Q2 | 27,708 | 3,062 | 215,754 | 0.95 (0.90,1.00) | 1.01 (0.97,1.07) | 1.01 (0.96,1.06) | 1.01 (0.96,1.06) | 1.01 (0.96,1.06) |
| Q3 | 27,706 | 3,166 | 215,484 | 0.98 (0.93,1.03) | 1.07 (1.02,1.12) | 1.06 (1.01,1.12) | 1.06 (1.01,1.12) | 1.07 (1.02,1.12) |
| Q4 | 27,707 | 3,525 | 212,489 | 1.11 (1.05,1.16) | 1.13 (1.08,1.19) | 1.13 (1.07,1.18) | 1.12 (1.06,1.17) | 1.11 (1.06,1.17) |
| *P* for trend | |  |  | <0.001 | <0.001 | <0.001 | <0.001 | <0.001 |

Model 1: Adjusted for age and sex

Model 2: Model 1+ body mass index, smoking status, regular exercise, and income

Model 3: Model 2+ antihypertensive medication, dyslipidemia medication, systolic blood pressure, total cholesterol, history of stroke, history of coronary artery disease, and history of chronic kidney disease

Model 4: Model 3 + mean FPG

PAD, peripheral artery disease; FPG, fasting plasma glucose; CV, coefficient of variation; SD, standard deviation; VIM, variability independent of the mean
